# Optimizing the clinical utility of four proposed criteria for a persistent and impairing grief disorder by emphasizing core, rather than associated symptoms

**DOI:** 10.1017/S0033291719000254

**Published:** 2020-02

**Authors:** Stephen J. Cozza, M. Katherine Shear, Charles F. Reynolds, Joscelyn E. Fisher, Jing Zhou, Andreas Maercker, Naomi Simon, Christine Mauro, Natalia Skritskaya, Sidney Zisook, Barry Lebowitz, Colleen Gribbin Bloom, Carol S. Fullerton, Robert J. Ursano

**Affiliations:** 1 Center for the Study of Traumatic Stress, Uniformed Services University of the Health Sciences, Bethesda MD 20814, USA; 2 School of Social Work, Columbia University, New York, NY, USA; 3 University of Pittsburgh, Pittsburgh, PA, USA; 4 University of Zurich, Zurich, Switzerland; 5 New York University, New York, NY, USA; 6 Mailman School of Public Health, Columbia University, New York, NY, USA; 7 University of California, San Diego, San Diego, CA, USA

**Keywords:** Accidental death, bereavement, combat death, complicated grief, persistent complex bereavement disorder, prolonged grief disorder, psychiatric nosology, suicide, violent death

## Abstract

**Background:**

Distinguishing a disorder of persistent and impairing grief from normative grief allows clinicians to identify this often undetected and disabling condition. As four diagnostic criteria sets for a grief disorder have been proposed, their similarities and differences need to be elucidated.

**Methods:**

Participants were family members bereaved by US military service death (*N* = 1732). We conducted analyses to assess the accuracy of each criteria set in identifying threshold cases (participants who endorsed baseline Inventory of Complicated Grief ⩾30 and Work and Social Adjustment Scale ⩾20) and excluding those below this threshold. We also calculated agreement among criteria sets by varying numbers of required associated symptoms.

**Results:**

All four criteria sets accurately excluded participants below our identified clinical threshold (i.e. correctly excluding 86–96% of those subthreshold), but they varied in identification of threshold cases (i.e. correctly identifying 47–82%). When the number of associated symptoms was held constant, criteria sets performed similarly. Accurate case identification was optimized when one or two associated symptoms were required. When employing optimized symptom numbers, pairwise agreements among criteria became correspondingly ‘very good’ (*κ* = 0.86–0.96).

**Conclusions:**

The four proposed criteria sets describe a similar condition of persistent and impairing grief, but differ primarily in criteria restrictiveness. Diagnostic guidance for prolonged grief disorder in International Classification of Diseases, 11th Edition (ICD-11) functions well, whereas the criteria put forth in Section III of Diagnostic and Statistical Manual of Mental Disorders, Fifth Edition (DSM-5) are unnecessarily restrictive.

## Introduction

There is consensus that a clinical condition of unremitting and functionally impairing grief exists in a highly-impacted minority of the population bereaved by natural causes (Lundorff *et al*., 2017), with higher rates (14–76%) for those suddenly or violently (by trauma or disaster) bereaved of young loved ones (Kristensen *et al*., 2012). Symptoms include persistent yearning, longing and sorrow with intense emotional pain, loneliness in the absence of the loved one, excessive avoidance of reminders, rumination over troubling aspects of the loss and prolonged difficulty regulating emotional pain, sometimes remaining years after the death and often going undetected (Forstmeier and Maercker, 2007; American Psychiatric Association, 2013; World Health Organization, 2015). This disorder, variably referred to as complicated grief (CG), persistent complex bereavement disorder (PCBD) or prolonged grief disorder (PGD), results in significant functional impairment, and has been distinguished from other mental disorders, including major depressive disorder and post-traumatic stress disorder, in its phenomenology (Prigerson *et al*., 1996) and treatment response (Shear, 2015; Shear *et al*., 2016).

Given this condition's impact on health and functioning (Lannen *et al*., 2008; Buckley *et al*., 2012) and its response to grief-specific interventions (Bryant *et al*., 2014; Shear, 2015), acceptable criteria must be accurate in identifying cases that are likely to respond to available treatment. In 1997, Horowitz and coworkers presented the first criteria for CG, and four sets of alternative criteria have since been proposed. Prigerson *et al*. (2009) derived criteria for PGD (PGD_PLOS_*) using item response theory and combinatorics analyzing (*The acronym PLOS refers to the peer-reviewed open access scientific journal published by the Public Library of Science since 2006.) data collected from a community-based sample of bereaved widows (*n* = 317) in the Yale Bereavement Study. CG criteria were derived from a large sample of patients (*n* = 782) whose chief complaint was prolonged and impairing grief (Shear *et al*., 2011; Simon *et al*., 2011). The Diagnostic and Statistical Manual of Mental Disorders, Fifth Edition (DSM-5) *Anxiety, Obsessive-Compulsive Spectrum, Posttraumatic, and Dissociative Disorders Work Group* (American Psychiatric Association, 2013) created provisional criteria for a newly-named disorder, PCBD, labeling it a ‘condition for further study’. Recently, the World Health Organization (WHO) workgroup for trauma and stress disorders developed a consensus guideline for a condition also called PGD (ICD-11_PGD_) proposed for inclusion in the upcoming International Classification of Diseases, 11th Edition (ICD-11) (World Health Organization, 2015). Despite their shared name, ICD-11_PGD_ differs from PGD_PLOS_ in important ways (Mauro *et al*., 2018).

Two previous publications examined the clinical utility of PCBD, PGD_PLOS_, and CG criteria. Cozza *et al*. (2016) conducted a large community survey study (*n* = 1732) of family members bereaved by US military deaths for more than 1 year. They identified those with clinically significant grief symptoms (cases, *n* = 260) persisting longer than 1 year, and those clearly free of those symptoms (non-cases, *n* = 675). The authors determined the conditional probability of accurate inclusion of cases and exclusion of non-cases using CG, PGD_PLOS_, and PCBD criteria among both groups. Findings showed that the probability of accurate case inclusion using PCBD and PGD_PLOS_ criteria was significantly lower (53% and 59%, respectively) than using CG (92%) criteria. All criteria accurately excluded non-cases. This study focused on accurate identification of cases and non-cases, and excluded from the analyses individuals with moderate levels of grief symptoms (ICG > 20, but <30; *n* = 797). We have since received questions from colleagues regarding the performance of the different criteria in this ambiguous, subthreshold group (Smid and Boelen, 2016; Maciejewski and Prigerson, 2017). Therefore, we have conducted the current analyses using the full data set.

Mauro *et al*. (2017) conducted a similar comparison of PCBD, PGD_PLOS_, and CG criteria performance in a clinical sample of bereaved individuals seeking help for impairing grief (*n* = 326) or for mood or anxiety disorders (*n* = 86). Despite notable differences in samples and some difference in determination of caseness, results were strikingly similar to Cozza *et al*. (2016). The conditional probability of case identification using PGD_PLOS_ and PCBD was 60% and 70% of cases, respectively, whereas, CG identified nearly all cases. All criteria excluded bereaved help-seeking individuals without persistent grief. Neither Cozza *et al*. (2016) nor Mauro *et al*. (2017) examined the performance of the proposed ICD-11_PGD_ guidelines. A separate case-controlled field study that examined the use of the ICD-11_PGD_ guideline by clinicians suggests it can be used reliably (Keeley *et al*., 2016).

Maciejewski *et al.* (2016) presented analyses of sensitivity, specificity, and positive predictive power of PCBD, ICD-11_PGD_, and CG criteria by reanalyzing the Yale Bereavement Study data while using their previously analyzed results as a criterion standard. Employing this questionably tautological method, they found PCBD criteria had high sensitivity, specificity, and positive predictive power. Of particular importance, in the absence of a ‘gold standard’, they mathematically derived a criterion standard that has never been tested among individuals who present with clinically significant grief symptoms beyond 1 year post-death. Additionally, their algorithm for the ICD-11_PGD_ guideline required more associated symptoms than recommended by the WHO proposal (World Health Organization, 2015). The authors concluded that PGD_PLOS_, ICD-11_PGD_, and PCBD criteria identified ‘a single diagnostic entity’, but that CG was substantively different (Maciejewski *et al*., 2016). Notably, the restrictiveness of Maciejewski *et al*.’s (2016) operationalized ICD-11_PGD_ criteria and their lack of a clinically relevant criterion standard are methodological limitations that call their conclusions into question.

Proposed criteria share similar core symptoms (e.g. yearning, longing and preoccupation), yet differ in the number of associated symptoms required to meet diagnostic threshold (i.e. six of 12 for PCBD; five of nine for PGD_PLOS_; two of eight for CG; and one of seven for ICD_PGD_; descriptions of required core and associated symptoms for each proposed criteria set are provided in online Supplementary Table 1). All criteria sets require that significant associated distress or impairment also be present. A confusing array of claims about differences among proposed criteria has made it difficult to develop consensus with respect to which are best suited to be employed by diagnostic systems, such as DSM-5 (Forstmeier and Maercker, 2007; Maciejewski and Prigerson, 2017; Reynolds *et al.*, 2017), which is primarily intended for use by clinicians. In contrast, we hypothesized that proposed criteria sets share more commonality than differences and that observed differences in their ability to identify clinically significant persistent impairing grief is likely to be accounted for by differences in criteria restrictiveness, as reflected in greater numbers of required symptoms. Supportive of this hypothesis, we previously reported that by reducing the number of required associated symptoms, the ability of PCBD criteria to identify cases was significantly improved (93%) while still accurately excluding 96% of non-cases (Cozza *et al*., 2016). This finding is consistent with Hyman (2010), who warned that ‘highly specified lists of operationalized criteria trades interrater reliability for the exclusion of a significant number of individuals who by other measures would be counted as affected (p. 166)’. We agree that highly restrictive criteria are likely to over-specify the disorder they intend to define, limiting their clinical utility rather than assisting clinicians in their day-to-day diagnostic and treatment decisions.

Accordingly, the objective of the current report is to examine similarities and differences in performance among proposed criteria sets using a series of analyses within our full community sample of bereaved military family members. We aim to inform the development of clinically meaningful consensus criteria for a persistent grief disorder to be included in DSM-5.1. Specifically, we hypothesized that different proposed criteria identify greater or lesser numbers of a single condition rather than different grief-related conditions (as suggested by Maciejewski *et al*., 2016). We further expected that criteria restrictiveness (i.e. numbers of required associated symptoms) determine differential criteria performance, and we explored what would be an optimal number of associated symptoms for use by clinicians.

## Method

### Study sample

Data were derived from the National Military Family Bereavement Study, a study of the impact of military service member death on family members (http://www.militarysurvivorstudy.org). Participants provided informed consent after receiving a description of the study and included surviving parents, spouses/partners, siblings, and adult children (some of whom may have been minors at the time of death) of service members in the US military (Army, Navy, Air Force, Marines, and Coast Guard) who died by all circumstances of death (i.e. combat, accident, suicide, homicide/terrorism, illness, undetermined) since 11 September 2001. Participants were recruited through grief support organizations, online advertisements, and word-of-mouth. Consistent with proposed criteria sets, only family members who completed assessments more than 1 year after the death were included in the current analyses (*N* = 1732).

### Measures

The following instruments were used in the present analysis:

(1) Complicated Grief Questionnaire (CGQ) is a slightly modified self-report version of the Structured Clinical Interview for Complicated Grief (Bui *et al*., 2015), which is a valid and reliable instrument that can be used to generate a diagnosis using any of the four proposed criteria. The CGQ differs from the Structured Clinical Interview for Complicated Grief in that it has 26 rather than 31 items and in utilizing a five-point Likert scale (0 = ‘never’; 1 = ‘rarely’; 2 = ‘sometimes’; 3 = ‘often’; and 4 = ‘very often’).
(2) Inventory of Complicated Grief (ICG) is a 19-item self-report measure of clinically impairing grief symptom severity (Prigerson *et al*., 1995). The ICG has been widely used as a screening tool to determine severity of clinically impairing grief. A cutoff score of ⩾30 (Cozza *et al*., 2016) has been used as a conservative threshold to identify clinically significant cases.
(3) Work and Social Adjustment Scale (WSAS) is a five-item, reliable, and valid self-report measure of functioning (Mundt *et al*., 2002). It has been used in several clinical populations with a reliable cutoff for moderate-to-severe clinical impairment ⩾20. Participants were specifically requested to rate how different areas of functioning ‘are impaired because of your grief’.

In addition, participants were asked to describe their demographics (e.g. age, gender, race), relationship to the deceased (i.e. parent, spouse/partner, sibling, or child), and cause of death of their military service family member (i.e. combat-related, accidental, suicide, homicide, terrorism, illness, or other).

### Determination of clinical and subthreshold samples

We employed two widely-used and well-validated measures (ICG and WSAS) to select clinical cases and subthreshold participants. Conditional probabilities of clinical case inclusion and subthreshold exclusion using the PCBD, PGD_PLOS_, and CG criteria, and the ICD-11_PGD_ guideline were then determined. Our multidimensional approach, using both a measure of grief symptom severity (ICG) and of grief-associated functional impairment (WSAS), to define clinical cases increases the likelihood that we identified a group of individuals with clinically significant symptoms requiring clinical intervention. Although there is considerable evidence that an ICG score >25 is associated with a range of negative outcomes (e.g. Prigerson *et al*., 1995, 1996, Boelen *et al*., 2003), we used a more rigorous definition of caseness (ICG ⩾30; which has also been employed in treatment studies) (e.g. Shear *et al*., 2016), to ensure inclusion of only those participants who were unequivocally disordered. Although clinically significant functional impairment has been associated with WSAS ⩾10, we required that cases endorsed WSAS ⩾20, which has been considered to indicate moderate-to-severe functional impairment in studies of mood, anxiety, and eating disorders (Mundt *et al*., 2002; Mataix-Cols *et al*., 2005; Tchanturia *et al*., 2013).

We used the multidimensional criterion of ICG ⩾30 and WSAS ⩾ 20 to divide the sample. We note that this threshold is conservative, and it is possible to have clinically significant symptoms below this threshold, but we believe by using this dividing point, we can test whether proposed criteria can adequately identify those in the sample who almost certainly are suffering from persistent impairing grief. It should be necessary (though not sufficient) for a clinically useful criteria set to identify these individuals. To determine whether this threshold affected criteria performance, we re-ran analyses with varying combinations of threshold requirements for caseness [i.e. ICG (⩾20; ⩾25; ⩾30) and WSAS (⩾15; ⩾20)].

### Applying criteria sets

Our sample included participants who had been bereaved more than 1 year from any cause (i.e. combat, accident, suicide, terrorism/homicide, illness, and unknown to participant), thus meeting time requirements for all criteria sets. Symptom requirements for each criteria set were assessed by matching CGQ item responses to the criterion requirements. Details about how we matched CGQ items to PCBD, PGD_PLOS_, and CG criteria sets are provided in Cozza *et al*. (2016). Table 1 shows how we matched CGQ items to the ICD-11_PGD_ guideline. As described in our previous work, individual symptoms were considered present if at least one of the matched Complicated Grief Questionnaire items was endorsed as being present ‘often’ or ‘very often’ (⩾3 on a 0–4 Likert scale using the following anchors: ‘never’, ‘rarely’, ‘sometimes’, ‘often’, or ‘very often’) in the last month. To rate impairment, we asked ‘Over the past month, how often have you had grief reactions at a level that interfere with your life?’ We considered the requirement for disorder-related functional impairment met for each criteria set if symptoms interfered at least weekly in the participant's life.

**Table 1.** CGQ item matching to the ICD-11 PGD guideline

ICD-11 PGD Guideline	CGQ Item Match
A persistent and pervasive grief response:	
1. characterized by persistent longing for the deceased OR persistent preoccupation with the deceased;	Strong feelings of yearning or longing for your loved one OR thoughts or images of your loved one that intrude on your activities or on your thoughts about other things
2. that is beyond expected social or cultural norms;	None
3. significantly impairs the person's function;	Over the past month, how often have you had grief reactions at a level that interfere with your life? (AT LEAST “weekly, fewer days than not”)
4. and is accompanied by intense emotional pain as evidenced by the presence of AT LEAST ONE OF THE FOLLOWING:	
*sadness*	Intense sorrow and emotional pain because your loved one is gone
*guilt*	Negative thoughts about yourself in relation to your loved one or the death (for example, thinking that you let this person down or thinking you can't manage without them)
*anger*	Bitterness or anger related to the loss
*denial or difficulty accepting the death*	Feelings of disbelief or feeling like you can't accept the reality that your loved one is really gone
*feeling one has lost a part of one's self*	Feeling that a part of you died with your loved one
*emotional numbness*	Feeling shocked, stunned, or emotionally numb because of the death
*difficulty in engaging with social or other activities*	Significant difficulty or reluctance to pursue interests or plan for the future because your loved one is gone OR Feeling alone or detached from other people because of your loss

### Statistical analysis

Demographic characteristics, participant relationship to the deceased service member, cause of death, ICG and WSAS mean scores, and percentages above cutoffs were reported for the study sample. The percentage of accurately identified participants who met threshold for caseness, and the percentage of accurately excluded subthreshold participants (including 95% confidence intervals) were determined for PCBD, PGD_PLOS_, and CG criteria, and the ICD-11_PGD_ guideline.

To illustrate whether criteria sets identified similar or different cases within the sample, we calculated *κ* statistics to examine agreement (with ‘poor’ agreement <0.20; ‘fair’ agreement 0.20–0.40; ‘moderate’ agreement 0.40–0.60; ‘good’ agreement 0.60–0.80; and ‘very good’ agreement 0.80–1.00) in identifying cases between pairs of criteria sets in the entire sample (without regard to ICG or WSAS score). In addition, we constructed a Venn diagram to show the comparable pattern of criteria case identification using the four proposed criteria sets applied to the entire sample.

In order to examine the contribution of criteria restrictiveness (as determined by the number of associated symptoms) to their performance, summary receiver operating characteristic (ROC) plots were created for all criteria sets. These plots demonstrated changes in accurate clinical case inclusion and accurate subthreshold exclusion as the number of associated symptoms varied from 0 to as many as 6.

All statistical analyses were performed using SAS 9.4 (SAS Institute Inc., Cary, North Carolina, USA).

## Results

### Demographic and loss characteristics

Demographic characteristics, relationship type, cause of death, and the distribution of the ICG and WSAS scores of the study sample are presented in Table 2 (*n* = 1732). Thirty-seven percent met ICG threshold ⩾30 and when the additional requirement of WSAS ⩾20 was imposed, 260 participants met clinical caseness (16%), with the remaining 1402 identified as subthreshold.

**Table 2.** Demographic characteristics of the study sample

Characteristics	Community sample^a^ (*N* = 1732)	
	*N* or mean	% or Std.
Age in years (mean and Std.)	47.3	13.1
Gender		
Male	341	19.7%
Female	1388	80.3%
Race		
White	1584	91.8%
Other	142	8.2%
Ethnicity		
Hispanic	107	6.4%
Non-Hispanic	1557	93.6%
Participant relation to deceased service member		
Parent	971	56.2%
Spouse	388	22.5%
Sibling	321	18.6%
Adult child	48	2.8%
Cause of death of deceased service member		
Illness	100	5.8%
Combat related	842	49.0%
Accident	289	16.8%
Suicide	227	13.2%
Homicide/terrorist attack	130	7.6%
Unknown cause to participant	131	7.6%
Time since death in years (mean and Std.)	5.1	2.7
1–1.9 years	259	14.9%
2–3.9 years	432	24.9%
4–5.9 years	389	22.5%
6–9.9 years	582	33.6%
10–13.9 years	70	4.0%
Inventory of Complicated Grief total score (ICG, mean and Std.)	25.3	15.1
ICG (% ⩾ 30)	622	36.9%
Work and Social Adjustment Scale total score (WSAS, mean and Std.)	10.5	10.5
WSAS (% ⩾ 20)	325	19.0%

Percentages do not reflect missing data

a Sample with time since death more than 1 year

### Criteria performance: accurate case inclusion and subthreshold exclusion

Percentages of accurate identification of clinical cases and exclusion of those below this threshold are provided in Table 3. The most restrictive DSM PCBD criteria identified only 47% of clinical cases, with PGD_PLOS_ only slightly higher (53%). Both DSM PCBD and PGD_PLOS_ accurately excluded 96% of cases below the threshold. CG and ICD-11_PGD_ had higher rates of accurate case identification (81% and 82%, respectively), and excluded 89% and 86% of subthreshold participants, respectively. These results did not appreciably vary when we modified the threshold for caseness (e.g. ICG ⩾20, 25, or 30 and WSAS ⩾15 or 20; see online Supplementary Table S2 for more details).

**Table 3.** Accurate inclusion of cases and exclusion of subthreshold participants by proposed criteria

	Accurate case inclusion (*N* = 260)			Accurate non-case/subthreshold exclusion (*N* = 1402)		
	Cases identified (*n*)	%	95% CI	Non-cases excluded (*n*)	%	95% CI
DSM-5 PCBD criteria	120	46.7	(40.6–52.8)	1339	96.3	(95.3–97.3)
PGD PLOS criteria	137	53.1	(47.0–59.2)	1334	95.8	(94.8–96.9)
CG criteria	210	81.4	(76.7–86.1)	1229	88.5	(86.9–90.2)
ICD-11 PGD guideline	212	81.5	(76.8–86.3)	1196	85.7	(83.8–87.5)

Percentages do not reflect missing data and are based on case threshold of ICG ⩾ 30 and WSAS ⩾20

### Agreement among criteria sets

**Fig. 1.**
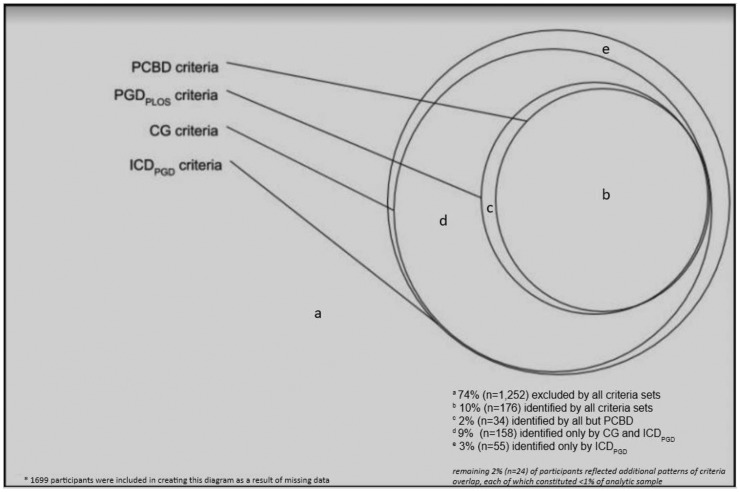
Participants identified by proposed criteria sets within community sample (*n* = 1732)*.

We constructed a Venn diagram (Fig. 1) to compare the pattern of case identification among the different criteria sets. Seventy-four percent of the entire sample (*n* = 1252) was excluded by all criteria. Ten percent of the sample (*n* = 176) was identified by all four criteria sets, which comprised virtually all cases identified by PGD_PLOS_ and PCBD criteria. All cases identified by PCBD and PGD_PLOS_ were also identified by both CG criteria and the ICD-11_PGD_ guideline. An additional 9% of the sample (*n* = 158) excluded by PGD_PLOS_ and PCBD were included by both CG and ICD-11, and 3% of the sample (*n* = 55) was identified only by the ICD-11_PGD_ guideline. Not surprisingly, *κ*s calculated to determine agreement between PCBD and PGD_PLOS_ showed ‘very good’ agreement (*κ* = 0.87), as did CG and ICD-11_PGD_ criteria (*κ* = 0.89). Additionally, all paired examinations demonstrated at least ‘moderate’ agreement with *κ*s ranging from 0.53 to 0.89. In particular, PGD_PLOS_ and CG evidenced ‘good’ agreement (*κ* = 0.64) indicating that these criteria are describing a very similar clinical syndrome (see online Supplementary Table S3 for details).

### Examination of the contribution of criteria restrictiveness to performance and agreement among criteria sets

**Fig. 2.**
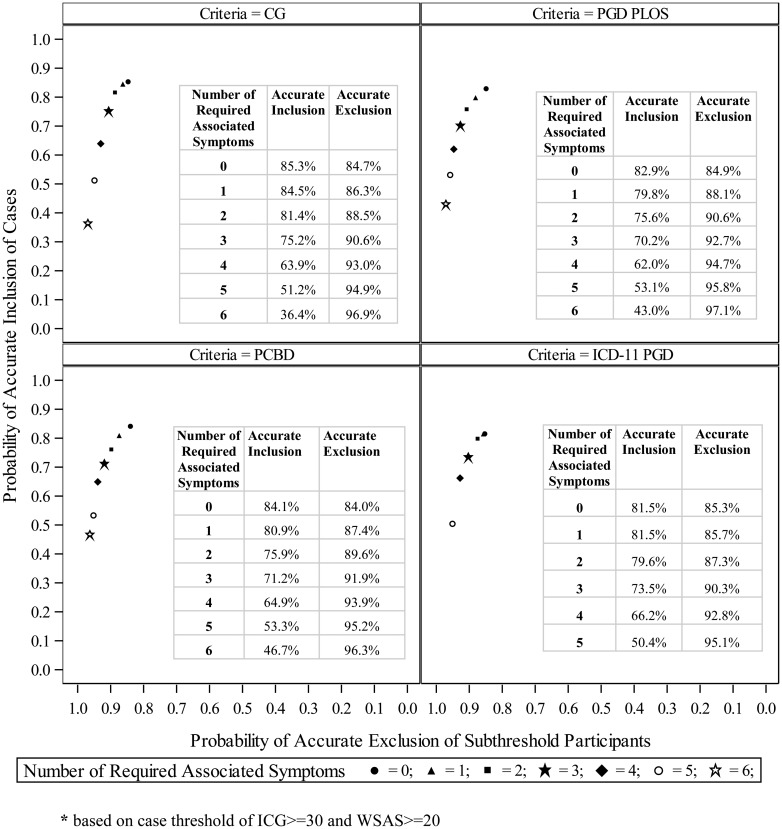
Receiver operating characteristics plots varying the number of required associated symptoms*.

Summary ROC plots (Fig. 2) demonstrated that varying the numbers of associated symptoms reduced the differences observed in criteria performance. When the number of required associated symptoms was held constant (i.e. at a fixed value from 0 and 6 associated symptoms), accurate inclusion of cases and exclusion of non-cases were nearly identical across criteria sets. Performance of all criteria was optimized when either one or two associated symptoms were required, and the level of agreement between all criteria similarly improved to ‘very good’ in all pairwise comparisons (*κ*s 0.86–0.96; see online Supplementary Table S4 for details).

## Discussion

Similar to our previous findings (Cozza *et al*., 2016), the less restrictive CG criteria set was more likely to accurately identify cases (81%) than either the PGD_PLOS_ or PCBD criteria sets (53% and 47%, respectively). The newly-assessed ICD-11_PGD_ performance was more similar to CG (82%) than PGD_PLOS_ or PCBD criteria sets. All proposed criteria demonstrated excellent ability to exclude subthreshold participants (86–96%). Results confirm and expand upon prior reports (Cozza *et al*., 2016; Maciejewski *et al*., 2016; Mauro *et al*., 2017) that compared criteria performance for a disorder of persistent and impairing grief. The multidimensional determination of caseness (using both ICG and WSAS) is a methodological strength of this study.

Better understanding of the similarities, as well as differences, among proposed criteria sets is a novel and important contribution. Similarities were evident in three ways. First, *κ*s indicated moderate-to-very good agreement among all criteria sets despite differences in their performance. Second, participants identified by the most restrictive criteria were also identified by the least restrictive criteria (see Fig. 1). In fact, the Venn diagram in Fig. 1 demonstrates that PCBD identified a subgroup of PGD_PLOS_, which identified a subgroup of CG, which identified a subgroup of ICD-11_PGD_. ROC plotting confirmed that this ‘Russian doll’ effect was due to the number of associated symptoms required. Third, when the number was held constant, all criteria sets performed similarly and agreement (*κ* comparisons) improved. This was true despite some differences in content and wording of defined associated symptoms across criteria sets. If criteria were truly identifying distinct disorders, as suggested by Maciejewski and Prigerson (2017), different participants would be identified by each criteria set, and differences in performance would not have been fully accounted for by the number of required symptoms.

This study has a number of limitations. The sample was comprised of family members bereaved by US military service deaths, the majority of which were sudden and violent. In addition, participants were volunteers and recruited through grief support organizations, online advertisements, and word-of-mouth rather than random sampling. Thus, we cannot address issues such as prevalence rates that depend upon random sampling. Neither older bereaved people nor non-violent deaths were well-represented. However, the results of Mauro *et al*. (2018), which used a sample of help-seeking individuals included older people who were bereaved mostly by natural causes, closely mirrored these findings, as well as our previous analyses (Cozza *et al*., 2016). Although our definition of caseness was rigorous, multi-dimensionally determined, and consistent with the evident literature, no clinical interviews were performed to confirm caseness. Although we described the ability of proposed criteria to differentiate persistent and impairing grief from depression in our prior report (Cozza *et al*., 2016), we did not examine the contribution of comorbid conditions in this current analysis. Because a gold standard has not yet been determined and because ours was not a random population sample, we believe it would be inappropriate to calculate sensitivity, specificity, or positive and negative predictive power of the proposed criteria with these data. These remain important goals for future work.

Our results clearly caution against overly restrictive criteria to define a disorder of persistent and impairing grief. We conclude that PGD_PLOS_ and PCBD criteria over-specify the disorder and, consistent with Hyman's (2010) warning, lead to misidentification of those suffering from a clinically disabling condition that could benefit from evidence-based treatments (Shear *et al*. 2005, 2014, 2016). In fact, clinical trial data confirm this conclusion. Research subjects who self-referred for a primary complaint of impairing grief, but were not identified by the restrictive PGD_PLOS_ and PCBD criteria, responded as positively to treatment as those who were diagnosed by these same criteria (Mauro *et al*., 2018).

Our findings indicate that emphasis on the presence of core symptoms of yearning/longing and/or preoccupation with the deceased, with inclusion of some associated symptoms in the context of meaningful functional impairment, optimizes each criteria set's clinical relevance and utility. Of those that have been proposed for implementation by diagnostic classification systems, the WHO's ICD-11_PGD_ guideline effectively identifies clinical cases, but the current DSM-5 PCBD criteria do not. Although associated symptoms can provide a broad description of the clinical expression of the disorder and are important for clinicians to recognize, a requirement of more than two of these symptoms diminishes PCBD criteria performance.

## Supplementary material

For supplementary material accompanying this paper visit http://dx.doi.org/10.1017/S0033291719000254. click here to view supplementary material
