# Supplementary material for: Optimizing the clinical utility of four proposed criteria for a persistent and impairing grief disorder by emphasizing core, rather than associated symptoms
Source: Psychol Med. 2019 Mar 4;50(3):438–45. doi: 10.1017/S0033291719000254 (PMC7025160; doi:10.1017/S0033291719000254)
Supplement: Supplementary file 1 [file S0033291719000254sup001.pdf]

Supplemental Table 1. Comparison of Core and Associated Symptoms across Proposed Criteria Sets

	DSM-5 (PCBD) criteria	PGD (PLOS) criteria	CG criteria	ICD-11 (PGD) guideline
**Core Symptoms**	Since the death, ***at least one of four symptoms*** experienced on more days than not and that have persisted at least 12 months after the death:<br>1. Persistent yearning/longing for Deceased<br>2. Intense sorrow and emotional pain in response to death<br>3. Preoccupation with deceased<br>4. Preoccupation with the circumstances of the death	Separation distress: ***The bereaved person experiences yearning*** (e.g., craving, pining, or longing for the deceased; physical or emotional suffering as a result of the desired but unfulfilled reunion with the deceased) daily or to a disabling degree	***At least one of four symptoms*** of persistent, intense, acute grief has been present for a period longer than is expected by others in the person's social or cultural environment:<br>1. Persistent intense yearning or longing for the person who died<br>2. Frequent intense feelings of loneliness or like life is empty or meaningless without the person who died<br>3. Recurrent thoughts that it is unfair, meaningless, or unbearable to have to live when a loved one has died or a recurrent urge to die in order to find or to join the deceased<br>4. Frequent preoccupying thoughts about the person who died (e.g., thoughts or images of the person intrude on usual activities or interfere with functioning)	***Longing for the deceased or persistent preoccupation with the deceased***
**Associated Symptoms**	Since the death***, at least six of twelve symptoms*** experienced more days than not and that have persisted for at least 12 months:<br>1. Marked difficulty accepting death<br>2. Experiencing disbelief or emotional numbness<br>3. Difficulty with positive reminiscing about the deceased<br>4. Bitterness or anger related to death<br>5. Maladaptive appraisals about oneself in relation to the deceased or the death (e.g., self-blame)<br>6. Excessive avoidance of reminders of the loss<br>7. A desire to die in order to be with the deceased<br>8. Difficulty trusting other individuals since the death<br>9. Feeling alone or detached from other individuals since the death<br>10. Feeling that life is meaningless or empty without the deceased or the belief that one cannot function without the deceased<br>11. Confusion about one's role in life or a diminished sense of one's identity<br>12. Difficulty or reluctance to pursue interests since the loss or to plan for the future	Cognitive, emotional and behavioral symptoms: ***The bereaved person must have five or more (of nine) symptoms*** experienced daily or to a disabling degree:<br>1. Confusion about on'es role in life or diminished sense of self (i.e., feeling that a part of oneself has died)<br>2. Difficulty accepting the loss<br>3. Avoidance of reminders of the reality of the loss<br>4. Inability to trust others since the loss<br>5. Bitterness or anger related to the loss<br>6. Difficulty moving on with life (e.g., making new friends, pursuing new interests)<br>7. Numbness (absence of emotion) since the loss<br>8. Feeling that life is unfulfilling, empty, or meaningless since the loss<br>9. Feeling stunned, dazed, or shocked by the loss	***At least two of eight symptoms are present*** for at least a month:<br>1. Frequent troubling rumination about circumstances or consequences of the death (e.g., concerns about how or why the person died or about not being able to manage without the loved one, thoughts of having let the deceased person down, etc.)<br>2. Recurrent feeling of disbelief or inability to accept the death, lieke the personcannot believe or accept that his or her loved one is really gone<br>3. Persistent feeling of being shocked, stunned, dazed, or emotionally numb since the death<br>4. Recurrent feelings of anger or bitterness related to the death<br>5. Persistent difficulty trusting or caring about other people or feeling intensely envious of others who have not experienced a similar loss<br>6. Frequently experiencing pain or other symptoms that the deceased person had or hearing the voice of or seeing the deceased person<br>7. Experiencing intense emotional or physiological reactivity to memories of the person who died or to reminders of the loss<br>8. Change in behavior due to excessive avoidance or the opposite, excessive proximity seeking (e.g., refraining from going places, doing things, or having contact with things that are reminder of the loss or feeling drawn to reminders of the person, such as wanting to see, touch, hear or smell things to feel lose to the person who died). (Sometimes people experience both of these seemingly contradictory symptoms.)	Accompanied by intense emotional pain as evidenced by the presence of ***at least one of the following:***<br>1. Sadness<br>2. Guilt<br>3. Anger<br>4. Denial or difficulty accepting the death<br>5. Feeling one has lost a part of one's self<br>6. Emotional numbness<br>7. Difficulty in engaging with social or other activities

Supplemental Table 2: Accurate Inclusion and Exclusion by Proposed Criteria with Varying ICG and WSAS Cutoffs

	**DSM-5 (PCBD) criteria**			**PGD (PLOS) criteria**			**CG criteria**			**ICD-11 (PGD) Guideline**	
	**Accurate Inclusion**	**Accurate Exclusion**		**Accurate Inclusion**	**Accurate Exclusion**		**Accurate Inclusion**	**Accurate Exclusion**		**Accurate Inclusion**	**Accurate Exclusion**
**WSAS>=15, ICG>=20**	32.4% (N=465)	98.1% (N=1201)		36.9% (N=465)	97.9% (N=1201)		63.4% (N=465)	93.4% (N=1201)		65.9% (N=465)	91.1% (N=1201)
**WSAS>=15, ICG>=25**	35.2% (N=427)	98.1% (N=1235)		40.1% (N=427)	97.9% (N=1235)		67.4% (N=427)	93.1% (N=1235)		68.8% (N=427)	90.3% (N=1235)
**WSAS>=15, ICG>=30**	39.6% (N=377)	98.1% (N=1285)		44.8% (N=377)	97.8% (N=1285)		72.2% (N=377)	92.2% (N=1285)		73.7% (N=377)	89.5% (N=1285)
**WSAS>=20, ICG>=20**	41.7% (N=295)	96.2% (N=1367)		47.4% (N=295)	95.8% (N=1367)		76.6% (N=295)	89.2% (N=1367)		76.9% (N=295)	86.3% (N=1367)
**WSAS>=20, ICG>=25**	43.1% (N=285)	96.3% (N=1377)		48.9% (N=285)	95.8% (N=1377)		78.0% (N=285)	89.1% (N=1377)		77.8% (N=285)	86.1% (N=1377)
**WSAS>=20, ICG>=30**	46.7% (N=260)	96.3% (N=1402)		53.1% (N=260)	95.8% (N=1402)		81.4% (N=260)	88.5% (N=1402)		81.5% (N=260)	85.7% (N=1402)

Percentages do not reflect missing data

Supplemental Table 3. Agreement (Kappa) between proposed criteria (N=1732)				
	**DSM-5 (PCBD) criteria**	**PGD (PLOS) criteria**	**CG criteria**	**ICD-11 (PGD) Guideline**
**DSM-5 (PCBD) criteria**				
**PGD (PLOS) criteria**	0.87			
**CG criteria**	0.58	0.64		
**ICD-11 (PGD) Guideline**	0.53	0.58	0.89	

Supplemental Table 4. Agreement (Kappa) between criteria with 1 or 2 required associated symptoms (N=1732)

Comparing criteria using 1 required associated symptoms

	DSM-5 (PCBD) criteria	PGD (PLOS) criteria	CG criteria	ICD-11 (PGD) Guideline
**DSM-5 (PCBD) criteria**				
**PGD (PLOS) criteria**	0.96			
**CG criteria**	0.92	0.91		
**ICD-11 (PGD) Guideline**	0.90	0.89	0.93	

Comparing criteria using 2 required associated symptoms

	DSM-5 (PCBD) criteria	PGD (PLOS) criteria	CG criteria	ICD-11 (PGD) Guideline
**DSM-5 (PCBD) criteria**				
**PGD (PLOS) criteria**	0.95			
**CG criteria**	0.89	0.88		
**ICD-11 (PGD) Guideline**	0.87	0.86	0.89	
